# Game Theory Model of Traffic Participants within Amber Time at Signalized Intersection

**DOI:** 10.1155/2014/756235

**Published:** 2014-12-15

**Authors:** Weiwei Qi, Huiying Wen, Chuanyun Fu, Mo Song

**Affiliations:** ^1^School of Civil Engineering and Transportation, South China University of Technology, Guangzhou 510641, China; ^2^School of Transportation Science and Engineering, Harbin Institute of Technology, Harbin 150090, China; ^3^Department of Automobile Service Engineering, Zhejiang Traffic Technician College, Jinhua 321000, China

## Abstract

The traffic light scheme is composed of red, green, and amber lights, and it has been defined clearly for the traffic access of red and green lights; however, the definition of that for the amber light is indistinct, which leads to the appearance of uncertainty factors and serious traffic conflicts during the amber light. At present, the traffic administrations are faced with the decision of whether to forbid passing or not during the amber light in the cities of China. On one hand, it will go against the purpose of setting amber lights if forbidding passing; on the other hand, it may lead to a mess of traffic flow running if not. And meanwhile the drivers are faced with the decision of passing the intersection or stopping during the amber light as well. So the decision-making behavior of traffic administrations and drivers can be converted into a double game model. And through quantification of their earnings in different choice conditions, the optimum decision-making plan under specific conditions could be solved via the Nash equilibrium solution concept. Thus the results will provide a basis for the formulation of the traffic management strategy.

## 1. Introduction

The research about urban road intersection is usually connected with the signal control, for example, adaptive signal control [1] and bus arrival time at signalized intersection [2]. And the running safety for road receives the attention of many scholars [3, 4]. In addition, Strauss et al. prove that motor-vehicle traffic is the main risk determinant at signalized intersection [5]. Jin et al. think that the pedestrians' road crossing behavior has negative impact on traffic safety [6]. Xie et al. show that the significance of the corridor-specific random effect and CAR effect revealed strong evidence for the presence of heterogeneity across corridors and spatial correlation among intersections [7, 8]. The vehicle conflict involving right-turn vehicles is an important part for the running safety at signalized intersections [9].

In 1960, Gazis, Herman, and Maradudin together put forward the notion of dilemma zone. The existence of dilemma zone usually results in red running and traffic accident, especially in the high speed traffic situation [10]. In other studies, many literatures have documented the impacts on driver's behavioral patterns due to the implementation of driver warning indicators, such as the studies on green signal countdown devices [11]. Amber light plays an essential role in the safety and efficiency of intersections; however, due to the lack of explicit definition of the function and the setting criterion for the amber light, a great deal of problems appears in movement of intersections [12, 13].

Factors such as approaching time to the stop line and the average retardation rate which related to drivers' behavior are studied by statistical methods abroad, which lay the foundation of the research on driving behavior characterization and signal control strategy during the amber light [14, 15]. For example, quantitative analysis on drivers' behavior characteristics at the moment when the amber light lightens up such as reaction time, braking time, and stop-passing decision are conducted by Rakha et al. [16]. Besides that, numerous famous scholars in traffic engineering and system engineering contributed a lot to the optimization of the amber light, and the research results of them are widespread and used in the area of urban traffic control and management [17–19].

Accompanied with the upsurge in the research of game theory, game models used for economics are applied to traffic engineering by plenty of domestic scholars, and excellent results are obtained in varied areas such as traffic congestion toll, drivers' decision-making behavior, and jaywalking phenomena [20]. It can be concluded that game theory is an effective way to solve the traffic participants' conflicting.

In this paper, in view of the lack of guidelines for amber light signal currently in China, the game theory, referring to domestic and foreign experience, is adopted to construct static and dynamic game models between drivers and traffic administration during the amber light of signal intersection. The decision that motives cancelling amber light in certain cities is revealed, and the decision based on the installation of amber light is illuminated; thus theoretical basis for the establishment of the corresponding rule of amber light is provided.

## 2. Decision-Making of Traffic Participants during Amber Light

As a complicated part of the road traffic system in city, intersection is much more complex than the other normal lane in road traffic system. There exist numerous traffic conflict points in the intersection. According to relevant research, the 32 conflict points (including 16 crossing conflicts, 8 confluence conflicts, and 8 diffluence conflicts) exist in a typical intersection. So drivers are facing up decision in each conflict, and any carelessness from drivers may result in an accident.

**Figure 1:**
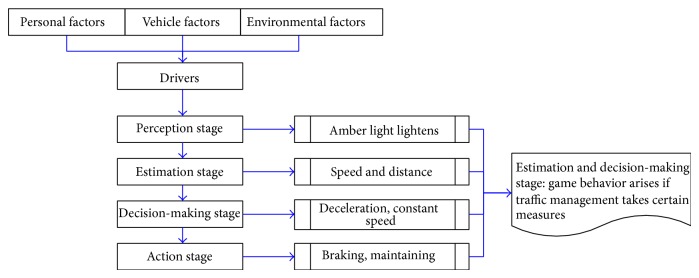
Drivers' decision process during amber light in road intersection.

Drivers are facing up decision-making all along in the process of driving, and one failed decision may lead to traffic conflict, even traffic accident. Proper decision can be vital when vehicles are approaching a signal intersection whose traffic signal is composed of red, green, and amber light. Decision during red and green light is simple: which is that red is the sign to stop, while green is to go ahead. However, during amber light, drivers should make a decision to slow down for stopping or pass through without deceleration, and the drivers' normal decision process is shown in Figure 1.

Based on the drivers' decision process in Figure 1, vehicles approach the signal intersection when the amber light lightens, and the drivers will make decision in accordance with their personal factors, vehicle factors, and environmental factors. Since different drivers have different understandings of the three factors mentioned above, their decisions are different too. Besides, passing rules and relevant management measures for amber light varied between a city and another in China, thus leading to extremely complex quantitative analysis on drivers' decision behavior during amber light.

The factors of drivers' personal feelings and experience are ignored in this paper to simplify the game model. So the game model between drivers and traffic administration is constructed based on the assumption that both of them (drivers, traffic administration) are fully rational, and a new strategy for amber light management is discussed.

## 3. Static Game Models between Drivers and Traffic Administration

The game model for mixed strategy is constructed between drivers and traffic administration ignoring their decision sequence on amber light. Drivers and traffic administration are assumed to be fully rational, and their revenue functions (*Ω*_*i*_) are composed of time efficiency factor (*e*, *e* > 0) and safety efficacy factor (*s*, *s* > 0). Further assume that the probability for traffic administration to permit vehicles passing through during amber light is *p*, while the probability for traffic administration to forbid vehicles passing through during amber light is (1 − *p*). Then assume that the probability for drivers to choose passing through the intersection obeying the rules during the amber light is *q*, while the probability for drivers to choose passing through the intersection violating the rules (violating the rules means that the drivers, who do not fall into dilemma zone, choose passing the intersection forcibly and take serious conflicts with other vehicles during the amber time) during the amber light is (1 − *q*). Thus, the revenue matrix of the mixed strategy game model between drivers and traffic administration is shown in Table 1 based on the above assumption, and the logical interpretations for the value of the revenues are shown in Table 2. The revenue functions for drivers and traffic administration are shown, respectively, in formula (1) and formula (2).

Time efficiency factor means the benefit that drivers or traffic administration gained in the travel time after they made a combined decision which aimed at amber light.

Safety efficacy factor means the safety benefit that drivers or traffic administration gained after they made a combined decision which aimed at amber light. Consider

(1) Ωmanagerp,q=pqe−s2+p1−q2e−2s +(1−p)q(2s−2e) +1−p1−qs−e2,

(2) Ωdriverp,q=pq(s−e2)+p(1−q)(2e−2s) +(1−p)q(2s−2e) +1−p1−qe−s2.

First order conditions of formula (1) and formula (2) are as follows:

(3) $$\begin{array}{c} \frac{\partial \Omega_{\text{manager}}}{\partial p}=\left(\frac{1}{2}e+\frac{1}{2}s\right)q-\left(3s-\frac{5}{2}e\right)=0,\\ \frac{\partial \Omega_{\text{driver}}}{\partial q}=\left(\frac{1}{2}e+\frac{1}{2}s\right)p-\left(3e-\frac{5}{2}s\right)=0. \end{array}$$

According to formula (3), Nash equilibrium of the mixed strategy game model can be solved as follows:

(4) $$\begin{array}{c} p^{*}=\frac{6e-5s}{e+s},\\ q^{*}=\frac{6s-5e}{e+s}. \end{array}$$

It can be concluded from formula (4) that the Nash equilibrium of the mixed strategy game model is closely related to time efficiency *e* and safety efficacy *s* and then game phenomenon between drivers and traffic administration in different conditions can be discussed according to the value of  *e*  and *s*. According to the basic definition of probability, the value range of *p* ^*^ and *q* ^*^ is from 0 to 1 (namely, 0 ≤ *p* ^*^ ≤ 1 and 0 ≤ *q* ^*^ ≤ 1). And the inequalities can be deduced as follows combined with formula (4):

(5) $$\begin{array}{c} 0\leq \frac{6e-5s}{e+s}\leq 1,\\ 0\leq \frac{6s-5e}{e+s}\leq 1. \end{array}$$

Formula (5) can be deduced as formula (6):

(6) $$\begin{array}{c} \frac{5}{6}s\leq e\leq \frac{6}{5}s,\\ \frac{5}{6}e\leq s\leq \frac{6}{5}e. \end{array}$$

Thus, the results can be discussed as follows:

1. if *e* = (5/6)*s*, then *p* ^*^ = 0 and *q* ^*^ = 1;
2. if *s* = (5/6)*e*, then *p* ^*^ = 1 and *q* ^*^ = 0.

Benefits for both sides of the game model in some special conditions are discussed as follows: since there are two factors in the revenue functions that are time efficiency and safety efficacy, it is difficult to judge which factor is more important; therefore it can be assumed that *e* = *s* = *c* (*c* is constant and *c* > 0) and thus a new revenue matrix can be established as in Table 3. According to the method of Nash equilibrium, the optimum solution of the matrix is *p* ^*^ = 0.5 and *q* ^*^ = 0.5.

On the premise that time efficiency *e* and safety efficacy *s* are of equal importance, results indicate that the probabilities for drivers and traffic administration to choose their own two strategies are the same. That is to say, the strategy (forbidding vehicles passing through intersections), proposed by traffic administration, is theoretically reasonable but not optimal. Since probabilities of the four strategies are the same when the revenue is optimal, it cannot be proved which strategy is more reasonable via this static game model; therefore a dynamic game model needs to be constructed to seek the best strategy profile in next part.

## 4. Dynamic Game Models between Drivers and Traffic Administration

The specific implementation process of traffic signal management policy is that traffic administration makes a plan at first, and then drivers make corresponding decisions in accordance with the plan. Traffic administration and drivers sequentially make their decision; hence it is necessary to construct a dynamic game model of perfect information.

**Figure 2:**
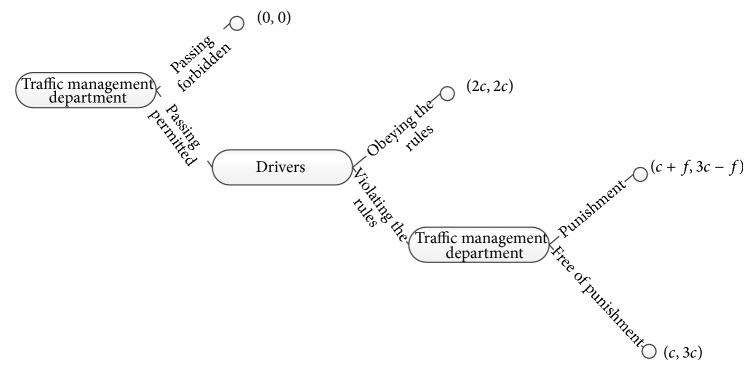
The extended dynamic game model between drivers and traffic administration.

Assume that both sides (drivers and traffic administration) of the game can fully understand their revenues and the game process; thus the extended dynamic game model between drivers and traffic administration can be established as in Figure 2. The revenues are shown in Table 4 (where *e* indicates time efficiency and *s* indicates safety efficacy, while *f* (*f* > 0) indicates illegal punishment). The logical interpretations for the value of the revenues are shown in Table 5. Referring to the above, the revenues can be simplified as shown in Table 6 assuming *e* = *s* = *c* (*c* > 0).

“Backward induction” is a method to solve the Nash equilibrium for game model of complete and perfect information. Operations at the end of the game are considered at first, and the optimal operations of participators in each case are determined in this method. Then these operations are regarded as the given future operations and continue to reverse forward in accordance with the time; thus the optimal operations of each participator are confirmed again until the beginning of the game process, as shown in Figure 2.

(1) The third stage is the traffic administration decision-making stage. In this stage, the traffic administrations make comparison of different revenues: since *c* + *f* > *c* always stand up, the traffic administrations must choose the strategy of punishment in the third stage and then reverse into the drivers' decision-making stage.

(2) The second stage is the drivers' decision-making stage. In this stage, drivers make decision and make comparison of different revenues: if 3*c* − *f* > 2*c* (namely, *f* < *c*), drivers will choose to violate the rules to pass the intersection irregularly, while if 3*c* − *f* < 2*c* (namely, *f* > *c*), drivers will choose to obey the rules to pass the intersection regularly and then reverse into the traffic administration decision-making stage.

(3) The first stage is the traffic administration decision-making stage. In this stage, the traffic administration makes decision and makes comparison of different revenues: if *f* < *c*, the traffic administrations will compare (*c* + *f*) with 0; if *f* > *c*, the traffic administrations will compare 2*c* with 0, which means that the rational traffic administration should choose the strategy permitting vehicles to pass the intersection during the amber light regardless of the value of *f*.

The calculation results of the dynamic game indicate that the strategy (forbidding vehicles to pass the intersection) during the amber light is against the principles of economics, and it is not conducive to the optimal revenues between the traffic administration and the drivers. The ideal strategy profile is that the traffic administrations permit vehicles to pass the intersection during the amber light and drivers pass through the intersection regularly.

## 5. Conclusions

The model between drivers and traffic administration is established by game theory based on the benefit-tending characteristics of the drivers and traffic administrations, incorrect inducement for traffic administration to take strategy that forbids vehicles to cross the intersection during the amber light, and the reason why drivers tend to cross the intersection irregularly is revealed. Specific conclusions are as follows.

(1) The revenue function for drivers and traffic administration is composed of time efficiency and safety efficacy and the probability that they choose certain behavior is determined by both the time efficiency and safety efficacy. Static and dynamic game model can be established separately considering whether there is an order for both of them to make decision.

(2) Traffic management strategy is always set in advance to drivers' behavior, and traffic administration and drivers usually make decisions in sequence. Thus it can be concluded from the dynamic game model that the best strategy for traffic administrations is to permit vehicles to cross the intersection during the amber light. While drivers' optimal strategy is related to penalty *f* based on the dynamic game models, the higher the value for *f* is, the more apt the drivers are to cross the intersection by obeying the rules. That is to say, traffic administrations can reduce the risk of drivers' illegal activities via the strategy (vehicles are permitted to cross the intersection during amber light via increasing the value of punishment on jaywalking phenomena).

(3) The game model between the traffic administration and drivers is established on the basis of the assumption that both of them are perfectly rational, and the revenue function for drivers and traffic administration is assumed to be composed of time efficiency and safety efficacy; therefore the theoretical analysis results may be different from the actual situation. It can be concluded that the focus of future study is how to lower the threshold of the assumptions and introduce multiple factors to represent the revenue function, so as to be more close to actual situation.

(4) The dilemma zone has long been considered as an issue for signalized intersection, especially for high speed signalized rural highways. However, it might not be the case for high-volume urban intersections where the speed is usually lower and stopping distance could usually be provided. So the game theory model for urban road intersection is established without considering the condition that drivers maybe fall in the dilemma zone during amber time. That is to say, the game theory model in this paper is applicable to the intersections in city, but not high speed signalized rural highways.

## Figures and Tables

**Table 1:** Revenue matrix of mixed strategy game model between drivers and traffic administration.

Revenue matrix 1			Drivers	
			Obeying the rules while passing	Violating the rules while passing
			*q*	1 − *q*
Traffic administration	Passing permitted	*p*	*e* − *s*/2, s − *e*/2	2*e* − 2*s*, −2*s* + 2*e*
	Passing forbidden	1 − *p*	−2*e* + 2*s*, 2*s* − 2*e*	−*e*/2 + *s*, −*s*/2 + *e*

**Table 2:** Logical interpretations for the value of the revenues.

Strategy profile	Revenue	Logical interpretations
Passing permitted, obeying the rules	(*e* − *s*/2, s − *e*/2)	When traffic administration permits passing and drivers obey the rules while passing the intersection during the amber light, which can improve the utilization efficiency of the green light, and traffic administration obtains the time efficiency *e*, while, compared with “passing forbidden” situation, they lose half of the safety efficacy *s*/2; on the other hand, drivers obtain the safety efficacy *s*, while, compared with “violating the rules” situation, they lose half of time efficiency* e*/2.

Passing permitted, violating the rules	(2*e* − 2*s*, −2*s* + 2*e*)	When traffic administration permits passing but drivers violate the rules while passing the intersection during the amber light, the traffic administration obtains higher time efficiency 2*e* but loses more safety efficacy 2*s*. The drivers obtain higher time efficiency 2*e *but lose more safety efficacy 2*s *as well.

Passing forbidden, obeying the rules	(−2*e* + 2*s*, 2*s* − 2*e*)	When traffic administration forbids passing and drivers obey the rules while passing the intersection during the amber light, which reduces the utilization efficiency of the green light, traffic administration loses higher time efficiency 2*e *but obtains more safety efficacy 2*s*. The drivers obtain more safety efficacy 2*s* but lose higher time efficiency 2*e*, compared with situation when drivers violate the rules.

Passing forbidden, violating the rules	(−*e*/2 + *s*, −*s*/2 + *e*)	When traffic administration forbids passing but drivers violate the rules while passing the intersection during the amber light, traffic administration obtains safety efficacy *s* but loses half of time efficiency* e*/2, while drivers obtain time efficiency *e *but lose half of the safety efficacy *s*/2.

**Table 3:** Simplified revenue matrix of mixed strategy game model between drivers and traffic administration.

Revenue matrix 2			Drivers	
			Passing through	Stop and wait
			*q*	1 − *q*
Traffic administration	Passing permitted	*p*	*c*/2, *c*/2	0, 0
	Passing forbidden	1 − *p*	0, 0	*c*/2, *c*/2

**Table 4:** Revenues of dynamic game between drivers and traffic administration.

Serial number	Game strategies		Revenues	
	Traffic administration	Drivers	Traffic administration	Drivers
1	Passing forbidden	—	0	0
2	Passing permitted	Obeying the rules	*e *+ *s *	*e *+ *s *
3	Passing permitted, punishment	Violating the rules	*e *+ *f *	3*e *− *f *
4	Passing permitted, free of punishment	Violating the rules	*e *	3*e *

**Table 5:** Logical interpretations for the value of the revenues.

Serial number	Game strategies		Logical interpretations	
	Traffic administration	Drivers	Traffic administration	Drivers
1	Passing forbidden	—	Vehicles are forbidden to pass during amber light, so no revenues were produced.	Vehicles are forbidden to pass during amber light, so no revenues were produced.

2	Passing permitted	Obeying the rules	Vehicles are permitted to pass during amber light, safety of each driver is protected, and the utilization efficiency of the green light is guaranteed; the traffic capacity of the intersection is improved. Safety efficacy and time efficiency can both be possessed by the traffic administration.	Vehicles are permitted to pass during amber light, each driver can make full use of green time and obtain adequate decision-making and operation time during the amber light, and thus drivers acquire both safety efficacy and time efficiency.

3	Passing permitted, punishment	Violating the rules	Vehicles are permitted to pass during amber light, the utilization efficiency of the green light is guaranteed, and the traffic capacity of the intersection is improved. Jaywalking phenomenon arises and punishment measures are taken by traffic administration, which provide time efficiency and penalty for traffic administration.	Vehicles are permitted to pass during amber light, and each driver can make full use of green time; however, drivers commit jaywalking to pursue higher time efficiency and thus drivers obtain higher time efficiency and minus punishments value.

4	Passing permitted, free of punishment	Violating the rules	Vehicles are permitted to pass during amber light, the utilization efficiency of the green light is guaranteed, and the traffic capacity of the intersection is improved. Jaywalking phenomenon arises, but there are no punishment measures taken; thus the traffic administration obtains time efficiency only.	Vehicles are permitted to pass during amber light, and each driver can make full use of green time; however, drivers commit jaywalking to pursue higher time efficiency, and thus drivers obtain higher time efficiency and avoid the punishment.

**Table 6:** Simplification of the revenues.

Serial number	Game Strategies		Revenues	
	Traffic administration	Drivers	Traffic administration	Drivers
1	Passing forbidden	—	0	0
2	Passing permitted	Obeying the rules	2*c *	2*c *
3	Passing permitted, punishment	Violating the rules	*c* + *f *	3*c* − *f *
3	Passing permitted, free of punishment	Violating the rules	*c *	3*c *
